# Membrane physiology and biophysics—Another milestone

**DOI:** 10.3389/fphys.2022.1081004

**Published:** 2022-11-25

**Authors:** Christoph Fahlke

**Affiliations:** Institute of Biological Information Processing, Molekular- und Zellphysiologie (IBI-1), Forschungszentrum Jülich, Jülich, Germany

**Keywords:** physiology, ion channel, transporter, channelopathies, transporteropathies

The *Membrane Physiology and Membrane Biophysics* section of *Frontiers in Physiology* was launched in 2010 by Mario Diaz of the University of La Laguna (Tenerife), who served as Specialty Chief Editor until I took over the position in 2020. *Frontiers in Physiology–Membrane Physiology and Membrane Biophysics* published its first article in October 2010, and this editorial celebrates the growth and maturation of the section as we publish our 600th article.

From its beginning, *Frontiers in Physiology–Membrane Physiology and Membrane Biophysics* has focused on the architecture, function, and cellular roles of biological membranes (Diaz, 2010; Fahlke, 2020). Biological membranes are barriers both between cells and between different cellular compartments and define cells and their organelles as separate entities. They are surprisingly simple in their basic design, consisting of lipid bilayers with integrated and associated membrane proteins. They can self-organize in an aqueous environment and flexibly change their form depending on external and internal forces. The low dielectric constant of lipid bilayers means that the movement of only a few ions across a membrane can generate a significant electrical potential gradient and, thus, form an electrical signal. This property makes electrical signaling both fast and energy efficient, allowing the rapid propagation and processing of information in excitable and non-excitable tissues. Biological membranes are both simple in design and easily adapted to perform a variety of functions. Lipid bilayers are almost perfect isolators, and the integration of transport proteins in biological membranes can confer selective permeability for different cations or anions or the ability for active transport to generate concentration gradients across the membrane.

Biological membranes are fascinating because of their simplicity and functional uniqueness as well as their high physiological importance. Cells communicate with each other at their plasma membranes, and many regulatory pathways are based on the membrane binding of transmitters and the consequent integration and processing of signals at these membranes. Hardly any cellular functions do not involve membrane transport or membrane signaling. The major importance of membrane physiology is illustrated by the variety of human diseases caused by dysfunctional membrane transport processes. An increasing number of channelopathies (i.e. human diseases caused by dysfunctional ion channels) have been identified in recent years (Wang et al., 1993). Pathological changes in transporter function are being increasingly recognized as the pathomechanism of human diseases (Jen et al., 2005; Bailey et al., 2011; Kovermann et al., 2022), and we expect transporteropathies to become an important topic in membrane physiology, as well as in organ physiology and clinical medicine.

From its beginning, our section has studied biological membranes at multiple scales, ranging from understanding their function at atomic resolution to detailed analyses of subcellular and cellular membrane signaling and on to their roles in pathogenesis. We have not restricted ourselves to any particular organ of the mammalian body, but have instead welcomed contributions on membrane research from researchers working in all branches of the animal and plant kingdoms. We are equally interested in unicellular organisms as in complex higher animals, and invite submissions based on both experimental and theoretical studies. We believe that understanding the mechanisms and consequences of membrane dysfunction provides important steps toward treating or correcting human diseases while also revealing normal membrane function.

So far, our section has hosted a total of 85 Research Topics. The diversity of scientific areas addressed in these Research Topics illustrates the breadth of our scientific interests. Some of these were very well received, as judged by the number of downloaded papers or citations. Among those were Research Topics about novel pathways for cell-to-cell communication (*Extracellular microvesicles and nanotubes in the brain: understanding their nature and function in cell-to-cell communication, their role in transcellular spread of pathological agents and their therapeutic potential*), about particular membrane transport processes with special impact for pathophysiology and medicine (*Regulation of red cell life-span, erythropoiesis, senescence and clearance*, *Ion transport in cell cycle and cancer,* and *Mesothelial physiology and pathophysiology*)*,* but also with biophysical (*Hidden secrets and lessons from the crystal structures of integral membrane proteins: channels, pumps and receptors*, and *Ligand recognition and regulation of ion channel proteins*) or physiological focuses (*Acid-base sensing and regulation: Molecular mechanisms and functional implications in health and disease*, and *Gap junctional communication in health and disease*). Other Research Topics, albeit of comparable impact for physiology and of similar scientific excellence, raised less interest. However, *Frontiers in Physiology–Membrane Physiology and Membrane Biophysics* does not focus only on mainstream research. We are convinced that studying membrane signaling in biological systems whose importance are not yet fully appreciated will provide novel and important insights, and we are determined to give a platform to these research areas.

Currently, around 80% of our papers are published as part of Research Topics, and the articles with highest numbers of uploads are mostly part of successful Research Topics (Bellingham et al., 2012; Chivet et al., 2012; Frühbeis et al., 2012; Hicks et al., 2012; Spuch et al., 2012; Leanza et al., 2013; Mairbäurl, 2013; Martin et al., 2013; Yang and Brackenbury, 2013; Clausen et al., 2017; Han et al., 2017). Although our Research Topics are an important method to disseminate scientific findings, we hope that our section will also be able to increase the percentage of individual submissions in the future. *Frontiers in Physiology–Membrane Physiology and Membrane Biophysics* is currently managed by 82 associate editors and 448 review editors from all over the world, from Postdocs to Laboratory heads, working in both academia and industry. We aim to provide a fair and critical review for all submissions, driven by curiosity and the ability to accept novel findings that overcome established concepts, without bias due to either personal rivalry or a preference for certain experimental approaches or scientific concepts. Our aim to improve diversity in age, gender, and geographical location in our editorial board will certainly help in reaching this goal.

In the last decade, our section has found its place in scientific publishing, and I have no doubt that it will continue to grow and excel. Our field has witnessed great progress in experimental as well as in computational approaches in recent past, and we expect these two separate fields to join into a close unity in future. We will utilize computational approaches to interpret experimental approaches and computational work to provide hypotheses that can be tested in experiments. Simulation techniques will also allow put individual transport processes into the context of cells, cell network and organ functions and to predict the consequences of their dysfunctions for the whole body. We will be able to predict ion concentrations and membrane potentials in cell organelles and whole cells using atomistic simulations, and these studies will certainly trigger simulations of cell excitability in cells and cell networks based on the structural dynamics of individual proteins. Thus far, we have hosted one Research Topic about this combination (*Combining computational and experimental approaches to characterize ion channels and transporters*), and I have little doubts that many that take advantage of both approaches will follow. Another important development for our future is the integration of individual studies on membrane transport at multiple scales into organ and whole body function. Such studies will be not only highly interesting for understanding normal body functions, but will revolutionize our view on human diseases. We will be able to envisage compensatory mechanisms in diseases that are triggered or caused by the dysfunction of individual proteins and predict novel therapeutic actions. A number of Research Topics in our section already address multi-scale studies (*The roles of ion channels in disease occurrence and development*, *New strategies for ion channel modulation in cardiovascular diseases*, *Ion channels and transporters in endocrine cells*, and *Ion channels in health and disease*). Such integrated view will certainly profit from the analysis of model organisms that are simpler and experimentally easier to modify as mammals, and we therefore expect that simple model organisms will continue to serve studying cell and organ physiology. Again, there are already Research Topics in our section that cover this important aspect (*Model organisms and experimental models in membrane physiology and membrane biophysics: Opportunities and challenges*). There will be also developments we are currently not able to foresee. *Frontiers in Physiology–Membrane Physiology and Membrane Biophysics* is looking forward to reporting novel findings, concepts and scientific approaches in the future and to continue contributing to the understanding of biological membranes as one of the most fascinating components of life.
